# *In situ* Scanning Electron Microscopy of Silicon Anode Reactions in Lithium-Ion Batteries during Charge/Discharge Processes

**DOI:** 10.1038/srep36153

**Published:** 2016-10-26

**Authors:** Chih-Yao Chen, Teruki Sano, Tetsuya Tsuda, Koichi Ui, Yoshifumi Oshima, Masaki Yamagata, Masashi Ishikawa, Masakazu Haruta, Takayuki Doi, Minoru Inaba, Susumu Kuwabata

**Affiliations:** 1 Department of Applied Chemistry, Graduate School of Engineering, Osaka University, 2-1 Yamada-oka, Suita, Osaka, 565-0871, Japan; 2 Department of Frontier Materials and Function Engineering, Graduate School of Engineering, Iwate University, 4-3-5 Ueda, Morioka, Iwate, 020-8551, Japan; 3 School of Materials Science, Japan Advanced Institute of Science and Technology, 1-1 Asahidai, Nomi, Ishikawa, 923-1292, Japan; 4 Department of Chemistry and Materials Engineering, Kansai University, 3-3-35 Yamate-cho, Suita, Osaka, 564-8680, Japan; 5 Department of Molecular Chemistry and Biochemistry, Doshisha University, 1-3 Tatara Miyakodani, Kyotanabe, Kyoto, 610-0321, Japan

## Abstract

A comprehensive understanding of the charge/discharge behaviour of high-capacity anode active materials, e.g., Si and Li, is essential for the design and development of next-generation high-performance Li-based batteries. Here, we demonstrate the *in situ* scanning electron microscopy (*in situ* SEM) of Si anodes in a configuration analogous to actual lithium-ion batteries (LIBs) with an ionic liquid (IL) that is expected to be a functional LIB electrolyte in the future. We discovered that variations in the morphology of Si active materials during charge/discharge processes is strongly dependent on their size and shape. Even the diffusion of atomic Li into Si materials can be visualized using a back-scattering electron imaging technique. The electrode reactions were successfully recorded as video clips. This *in situ* SEM technique can simultaneously provide useful data on, for example, morphological variations and elemental distributions, as well as electrochemical data.

Silicon is one of the most appealing next-generation anode active materials for future lithium-ion batteries (LIBs) with outstanding theoretical capacities (e.g., 3579 mAh g^−1^ for Li_15_Si_4_) nearly an order of magnitude greater than that of a conventional graphite battery (372 mAh g^−1^)^1^,^2^. However, the dramatic volume change (up to approximately 300%) experienced during charging/discharging, i.e., alloying/dealloying with Li, often leads to severe pulverization and the subsequent loss of electrical contact. In many cases, these mechanical phenomena result in a fatal capacity drop. This is a barrier to the practical application of Si anodes. Silicon nanostructuring is regarded as an effective strategy to relieve mechanical stress^2^. Several promising configurations have been proposed, such as nanoparticles^3^,^4^, nanowires^5^, nanotubes^6^, thin films^7^,^8^, nano-hairy structures^9^, and Si thin flakes (synonym: Si LeafPowder^®^)^10^,^11^. Of these, Si thin flakes are recognized as a well-balanced material because they can be readily stacked in layers without sacrificing the advantages associated with nanostructures; thus, they not only possess high, nanomaterial-like capacity and stable cyclabilities but also offer bulk-like tap density.

Ionic liquids (ILs), which are liquid salts at room temperature, have considerable potential as advanced electrolytes for LIBs owing to of their unique properties: wide electrochemical windows, negligible vapour pressures, nonflammability, and good thermal and electrochemical stability^12^,^13^. Several research groups have combined bis(fluorosulfonyl)amide (N(SO_2_F)_2_, FSA)-based ILs and Si anodes for the development of future LIB systems^14^,^15^,^16^,^17^. For example, Piper, *et al*. successfully combined these components into a LIB system^17^, which showed both remarkable capacity retention (>75% after 500 cycles) and an unprecedented coulombic efficiency (99.97% for the first 200 cycles). In the past decade, analytical approaches for studying an electrode reaction in a LIB cell, e.g., X-ray diffraction (XRD)^18^,^19^, atomic force microscopy (AFM)^20^, nuclear magnetic resonance (NMR)^21^, neutron depth profiling (NDP)^22^, X-ray tomographic microscopy (XTM)^23^, and transmission electron microscopy (TEM)^24^,^25^,^26^, have significantly improved. *In situ* scanning electron microscopy (*in situ* SEM), while receiving less attention in the battery researches as compared to TEM, will be a powerful and user-friendly analytical tool, because it is easy access to the setup for the *in situ* observation and SEM itself is a common analysis equipment. Indeed, we have published several articles on *in situ* SEM of electrochemical reactions in ILs^27^,^28^,^29^,^30^.

Herein, we describe *in situ* SEM techniques for the real-time visualization of Si active materials in a full cell with practical battery components and configurations. In a demonstration cell using a nonvolatile IL electrolyte and different Si active materials, the morphological and elemental distribution variations in the Si anodes were successfully observed and captured during charge/discharge processes with recording the electrochemical data. The potential advantages of size- and shape-controlled Si materials (e.g., Si thin flakes) over conventional Si particles are discussed based on the information obtained through *in situ* SEM techniques.

## Results and Discussion

**Figure 1:**
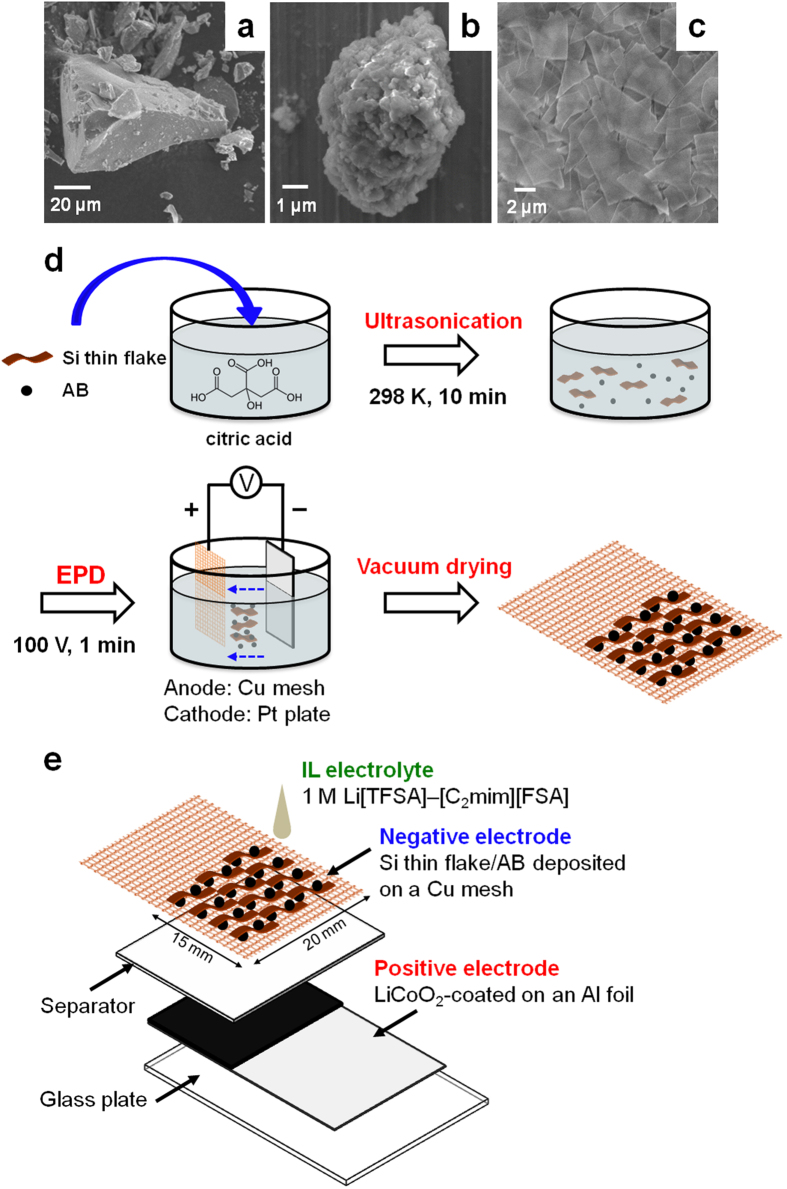
Schematic drawings of the Si anode preparation process and an *in situ* SEM observation cell. (**a–c**) SEM images of the Si active materials used in this study. The active materials were (**a**) Si microparticles, (**b**) Si nanoparticle aggregates, and (**c**) Si thin flakes. (**d**) Binder-free Si anode fabrication process via the electrophoretic deposition (EPD) method. Here, Si thin flakes were employed as the Si active material. (**e**) Illustration of a typical Li-ion battery system with a binder-free Si thin flake anode for *in situ* SEM.

The direct imaging of the electrochemical lithiation/delithiation behaviour of Si active materials during the charge/discharge process is of great importance in designing a future high-capacity anode with good cyclability. In this research, we employed binder-free Si electrodes prepared by an electrophoretic deposition (EPD) method as the anode. The surface electric charge state of the Si active materials and an acetylene black (AB) conduction supporting agent was modified by adding citric acid to a dry acetone bath. This enabled the codeposition of Si and AB onto a copper mesh current collector^31^. The active materials, which had different shapes and sizes, were Si microparticles, Si nanoparticle aggregates, and Si thin flakes, as shown in Fig. 1a–c. As an example, Fig. 1d depicts a fabrication process for a binder-free Si thin flake electrode. The conceptual cell setup for the *in situ* SEM equipment used in this research is illustrated in Fig. 1e. The electrodes and separator were located, and their configuration was quite similar to that found in a typical LIB. A nonvolatile IL electrolyte—1-ethyl-3-methylimidazolium bis(fluorosulfonyl)amide ([C_2_ mim][FSA]) containing 1.0 mol L^−1^ lithium bis(trifluoromethanesulfonyl)amide (Li[TFSA])—was selected as the electrolyte for the cell^32^. A photograph of the assembled cell is shown in Supplementary Fig. 1a. Supplementary Fig. 1b is a digital microscope image of the Si microparticle electrode. This electrode is covered with the IL electrolyte. Thus, the electrochemical lithiation/delithiation reactions of the Si active materials proceed in the areas where the active materials encounter the electrolyte. However, the electrolyte should be a very thin layer (up to approximately 100 nm) to obtain a clear *in situ* SEM image of the Si anode^33^,^34^,^35^. Otherwise, only a smooth IL surface will be observed, as shown in Supplementary Fig. 1c.

**Figure 2:**
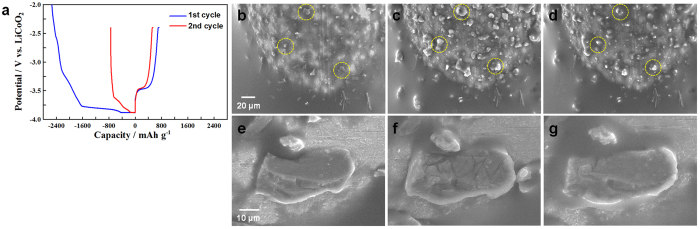
*In situ* SEM images of a Si microparticle anode during the 1st charge/discharge process. (**a**) The charge/discharge curves of a Si microparticle anode recorded in constant current and constant voltage (CC/CV) mode with cut-off voltages ranging between −3.88 V and −2.40 V (*vs*. LiCoO_2_). The CC rates for charge and discharge were 0.12 C. (**b–g**) *In situ* SEM images of the Si microparticles. (**b**,**e**) The initial state. (**c**,**f**) After the charge process. (**d**,**g**) After the discharge process.

Charge/discharge curves recorded at the Si microparticle anode are shown in Fig. 2a. At the 1st cycle, a substantial irreversible capacity was identified. Similar behaviour has also been reported by other research groups, and its underlying reason has been investigated^23^,^36^,^37^. Figure 2b–d show SEM images displaying the morphology variation in the Si microparticles during the 1st charge/discharge process. Typical several particles are surrounded by yellow circles. After the 1st charge process, the Si particles expanded considerably because of the formation of lithiated Si (Li_*x*_Si), but this expansion was not isotropic. This non-uniform expansion implies that lithiation initiates at a point between the Si particles and the current collector. The lithiated Si microparticles shrank during the 1st discharge process and did not fully recover to their original size. Enlarged images obtained from a different area during the same cycle are shown in Fig. 2e–g. Several cracks appeared during the charge process. Tensile stress in the external surface of the lithiated section is known to increase as lithiation proceeds^38^,^39^. The stress in the outer layer triggers fracturing that is directly related to capacity loss. Most of the cracks became nearly invisible as a result of shrinking back during the discharge process. Moreover, we succeeded in capturing this on video at the same place as Fig. 2e–g during the 2nd charge/discharge process (Movies 1 and 2 in the supplementary information). In Movie 1, severe volume expansion followed by a sudden reappearance of cracks was observed upon the 2nd charge process. Interestingly, the largest microparticle in the movie was moved by direct contact with a neighbouring small particle, and subsequently, the microparticle became electrochemically inactive, clearly indicating that it lost its electrical contact with the current collector (Movie 2). Two types of mechanical damage—fracturing of the Si microparticles and breakdown of the conductive network—resulting from the lithiation reaction were visually identified via *in situ* SEM. These types of damage mainly contribute to the enormous irreversible capacity observed in the first cycle, although other contributors should also be considered, such as solid-electrolyte interphase (SEI) formation resulting from the decomposition of the IL on the lithiated Si^14^,^17^,^40^.

We also observed the electrowetting behaviour of the IL electrolyte, which can be explained by the difference in the ion sizes of cations and anions^41^, during the charge process on an electron-microscopic scale. This information can be obtained only using this *in situ* technique. It will be useful information in designing the LIB cell with a bare minimum of the IL electrolyte. Compared to the *in situ* SEM image, the *ex situ* SEM image of the largest particle shown in Fig. 2g collected after rinsing the anode with diethyl carbonate (DEC) is completely different (Supplementary Fig. 2). Notably, the *ex situ* image includes artefacts that may arise during electrode recovery and rinsing. Therefore, *in situ* SEM is highly advantageous relative to *ex situ* observations.

**Figure 3:**
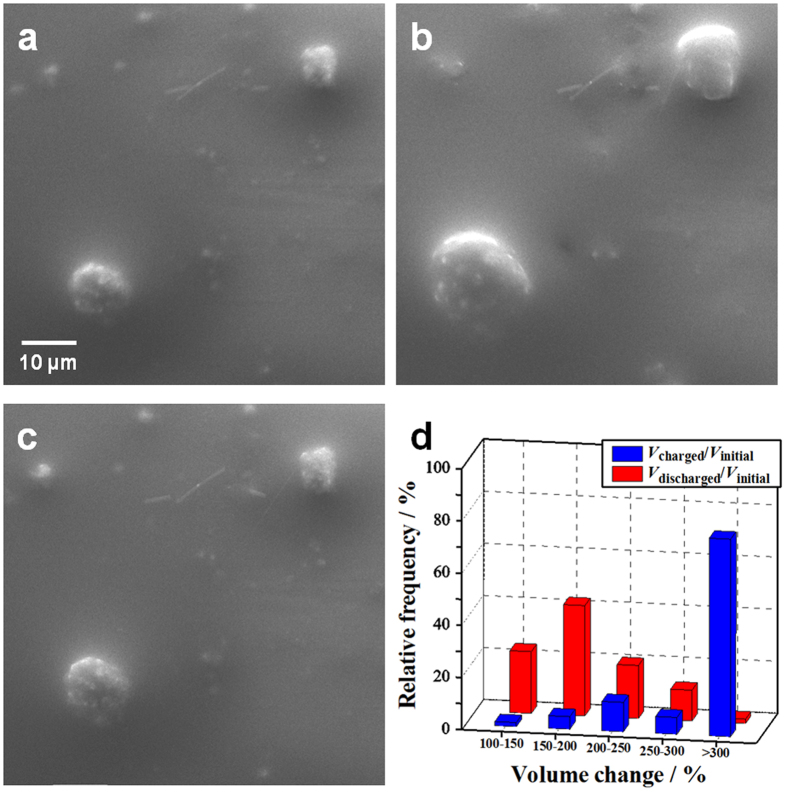
*In situ* SEM images of a Si nanoparticle aggregate anode (*D*_avg_ for aggregates: approximately 10 μm; *D*_avg_ for nano-Si particles: approximately 100 nm) during the 1st charge/discharge process. The charge/discharge conditions were 0.25 C (constant current mode) at cut-off voltages of −3.88 V and −2.40 V (*vs*. LiCoO_2_). (**a**–**c**) *In situ* SEM images of Si nanoparticle aggregates. (**d**) The relative frequencies as a function of the volume change in Si nanoparticle aggregates after (blue) the charge and (red) discharge processes relative to the initial volume.

For the Si nanoparticle aggregates, no cracks were identified during the 1st charge/discharge process (Fig. 3a–c). Most of the aggregates exhibited relatively isotropic volume expansion and shrinkage because the higher surface-to-volume ratio of the Si nanoparticle facilitates full lithiation. To quantify the lithiation behaviour of the Si nanoparticle aggregates, the volume variations (%) after the charge and discharge processes relatively to the initial volume—*V*_charged_/*V*_initial_ and *V*_discharged_/*V*_initial_, respectively—were estimated. We assumed that the height of each aggregate is half the sum of its width (*W*) and length (*L*). To reduce the error, the volume calculation was applied only to near-spherical aggregates (*L*/*W* < 1.30). The statistical analysis revealed that most Si particles after the charge process approached the theoretical value for Li_15_Si_4_ formation, approximately 370% (74.9 Å^3^ for Li_15_Si_4_ per Si atom and 20.0 Å^3^ for crystalline Si)^42^,^43^, and obvious irreversible volume alteration was observed after the discharge (Fig. 3d). The relative frequency of *V*_discharged_/*V*_initial_ peaked at a volume change of 150–200%. This is attributable to the fact that dense packing of Si nanoparticle aggregates loosened upon electrochemical cycling and nanoscale pores were generated through clustering of vacancies left behind by lithium-ion stripping^26^.

According to Griffith’s fracture criterion, cracking should occur only if the stored strain energy exceeds the surface energy of the new surface with cracks^2^,^38^,^39^. This model is also applicable to Si particles. The stress-relief volume and surface energy resistance relevant to crack growth depend on the Si particle size^38^. Various studies have been conducted to elucidate the critical Si particle size without lithiation-driven fracturing^38^,^39^,^44^,^45^,^46^. The critical particle sizes proposed to date range from hundreds of nanometres to 10 μm. For example, Kalnaus *et al*., on the basis of both experimental evidence and theoretical prediction, reported that the probability of mechanical failure sharply increases if the particle radius exceeds the proposed range^46^. Our *in situ* SEM results are in good agreement with the model vaguely proposed by a number of research groups.

**Figure 4:**
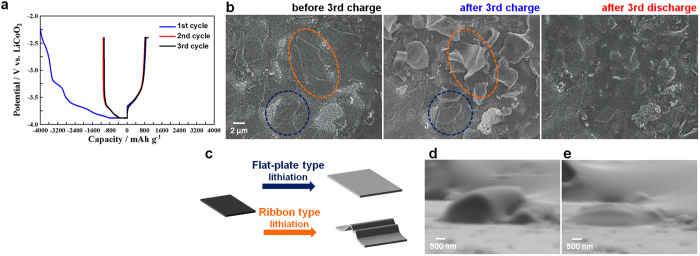
*In situ* SEM images of a Si thin flake anode during charge/discharge processes. (**a**) The charge/discharge curves of a Si thin flake anode recorded in constant current and constant voltage (CC/CV) mode with cut-off voltages ranging between −3.88 V and −2.40 V (*vs*. LiCoO_2_). The CC rates for the charge and discharge were 0.5 C. (**b**) *In situ* SEM images taken before and after the 3rd cycle. (**c**) Schematic drawings of two expansion models during the lithiation process revealed by *in situ* SEM. (**d**,**e**) *In situ* SEM images of a Si thin flake taken from the side after (**d**) the charge and (**e**) discharge processes.

Figure 4a shows the charge/discharge curves of a Si thin flake anode measured in constant current/constant voltage (CC/CV) and CC modes for charging and discharging, respectively, with cut-off voltages ranging between −3.88 V and −2.40 V (*vs*. LiCoO_2_). As described below, the discharge capacity retention stabilized after several cycles, although the irreversible capacity clearly appeared during the 1st cycling, similar to other Si active materials^7^,^8^,^14^,^31^. A series of SEM images captured from Movies 3 and 4 displaying the charge/discharge behaviour of a Si thin flake anode at the 3rd cycle are shown in Fig. 4b. As expected, the Si flakes expanded during charging. Surprisingly, however, two expansion processes were identified: a readily predictable flat-plate type expansion and a ribbon-type expansion (Fig. 4c). In the latter, the mechanical stress caused by the lithiation process appears to be spontaneously relieved by the flexible expansion. Indeed, we found no evidence of serious damage, e.g., fracturing and cracks, in the Si flakes during the cycle test. Figure 4d and e depict *in situ* SEM images from the side of a Si thin flake after charging and discharging. The mean thickness increased to approximately 150 nm from approximately 90 nm in the original flakes, and the lithiated Si flakes decreased to approximately 120 nm after the discharge. This irreversible volume change is due to the formation of nanoporous Si by delithiation from the resultant Li-Si alloy^26^. For reference, low-magnification *in situ* SEM images obtained during the 3rd cycle are shown in Supplementary Fig. 3a–h. The relevant charge/discharge curve is given in Supplementary Fig. 3i. These SEM images were captured at each point indicated by a-h in the charge/discharge curve.

**Figure 5:**
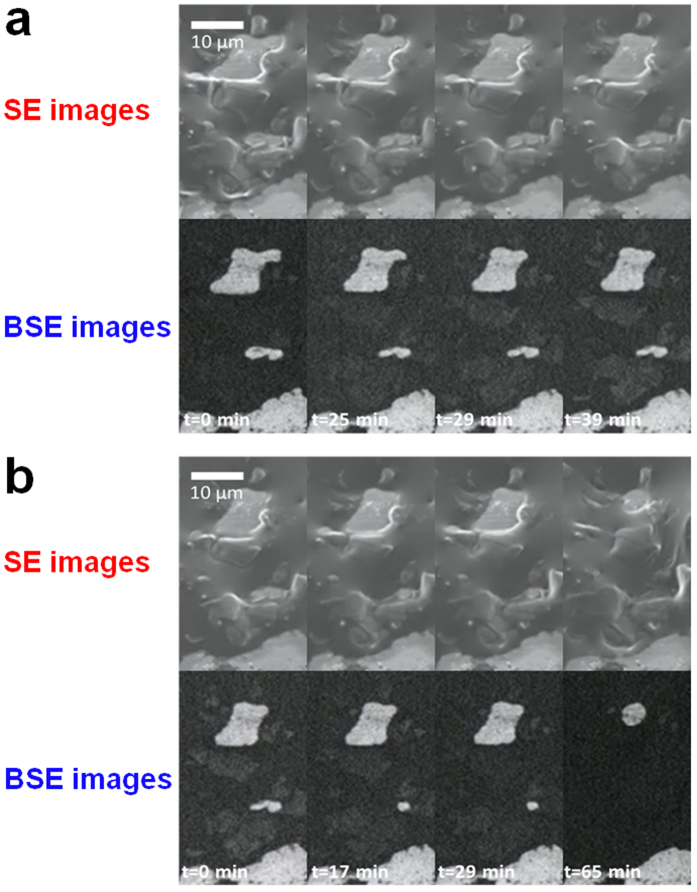
*In situ* secondary and backscattered electron images of a Si thin flake anode during (**a**) the 2nd discharge and (**b**) the 3rd charge processes. The charge/discharge process was conducted in constant current and constant voltage (CC/CV) mode with cut-off voltages ranging between −3.88 V and −2.40 V (*vs*. LiCoO_2_). The CC rates for the charge and discharge processes were 0.5 C.

In addition to the morphology variation in the Si active materials during the charge/discharge process observed by secondary electron (SE) imaging, we also used backscattered electron (BSE) imaging, which can provide dynamic information about elemental distributions. This technique enables the visualization of the lithiation/delithiation processes of Si materials by means of the composition contrast effect^47^, i.e., an element with a lower atomic number releases fewer backscattering electrons, thereby generating a dark image. Therefore, the BSE image of Si active materials is dark during the lithiation process because Li incorporates into them. To reveal a clear connection between morphological changes and lithium diffusion, in this investigation, both SE and BSE images were acquired simultaneously (Fig. 5 and Movie 5 and 6). During the charging process, the BSE image was darker because of the lower average atomic weight resulting from the lithiation process, but the brightness returned to its initial state after discharging. Interestingly, the brightness variation in each Si flake occurred uniformly over a short time, suggesting that quick lithiation/delithiation reactions occurred. Therefore, the remarkable charge/discharge characteristics of the Si thin flakes, which have been previously reported^10^,^11^, are attributable to their flexible nature and facile lithiation/delithiation kinetics, as visually demonstrated in this study.

**Figure 6:**
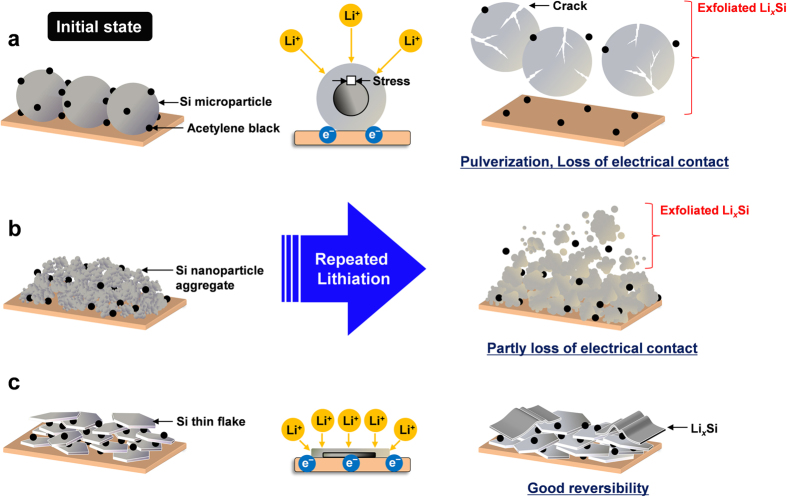
Schematic illustrations of morphology variation in Si active materials caused by charge/discharge processes. The Si active materials were (**a**) Si microparticles, (**b**) Si nanoparticle aggregates, and (**c**) Si thin flakes.

Figure 6 illustrates the lithiation process of Si active materials based on *in situ* SEM. An anode with Si microparticles suffered serious damage by pulverization and a conductive network breakdown at an early stage of lithiation (Fig. 6a). The use of Si nanoparticle aggregates mitigates the mechanical and electrical failures to some extent. However, as shown in Supplementary Fig. 4, the discharge capacity gradually decreased with the cycle number. Si nanoparticles exfoliate from the aggregates because of the insufficient strength of the interfacial bonds among the nanoparticles during continuous electrochemical cycling. Nevertheless, Si nanoparticles themselves have good potential as an anode active material for future LIBs^2^,^4^. This lithiation model is depicted in Fig. 6b. As for Si thin flakes, the Li diffusion length from a surface layer to the core is short, and its flexible nature can spontaneously relieve the stress caused by lithiation/delithiation processes (Fig. 6c). As a result, the Si anode exhibits better cyclability. As shown in Supplementary Fig. 4, the LIB cell employing Si thin flakes exhibited a stable discharge capacity after the initial few cycles. Furthermore, its retention was twice as high as that of a Si nanoparticle aggregate anode after 10 cycles.

Studying the morphological variations in different types of Si anode active materials in LIB cells during the charge/discharge process is directly related to the rational design of durable and high-capacity Si anodes. In this article, using our established *in situ* electron microscope techniques, we visually demonstrated that the fracture resistance and volume change characteristics of Si active materials show significant size and shape dependencies. This study revealed that Si thin flakes show an unexpected shape variation that spontaneously relieves the mechanical stress caused during charge/discharge processes. Additionally, the *in situ* electron microscope approach described here enables the simultaneous recording of three different types of information: electrochemical data, morphological variation process data, and dynamic information on the elemental distribution during the lithiation/delithiation of Si anode materials. If a SEM system for *in situ* observation is equipped with an extra X-ray detector, further useful information could be readily obtained to identify electrode reactions and parasitic reactions, including SEI formation, in LIB cells. This novel *in situ* analytical approach has great potential for emerging as a useful tool for tailoring high-capacity active materials, such as Si and Sn, and designing anodes for various next-generation IL-based energy-storage devices, including LIBs.

## Methods

### Fabrication of Si anodes

Si anodes were prepared by an EPD method as described in ref. 31. The solution used for the Si anode preparation was dry acetone containing 1.0-g L^−1^ Si active materials, 0.4-g L^−1^ AB (Strem Chemicals (USA)), and 1.0-g L^−1^ citric acid monohydrate (Wako (Japan)). The Si active materials used in this research were Si thin flakes (Si LeafPowder^®^ (Si-LP), thickness: 100 nm, lateral size: 4–5 μm, Oike & Co., Ltd., Kyoto (Japan)), Si microparticles (100 mesh, Nilaco Co. (Japan)), and Si nanoparticle aggregates (325 mesh, Rare Metallic Co., LTD. (Japan)). AB was added as a conduction supporting agent for the Si anodes. The solution was sonicated for 10 min prior to use. A platinum plate was used as the cathode, and copper mesh served as the anode. The electrodes were separated by 2 cm in the bath. The voltage difference between the two electrodes was set at 100 V, and the EPD duration was 15–120 sec. All the Si active material-deposited copper mesh electrodes were dried at 353 K under vacuum overnight to strengthen the adhesion of the Si materials.

### LIB cells for *in situ* SEM

The cell was composed of a binder-free handmade Si anode, a LiCoO_2_ cathode (3.0 mAh cm^−2^, Piotrek Co., Ltd. (Japan)), and a glass microfibre filter separator (GF/A, Whatman (UK)). The electrolyte was [C_2_ mim][FSA] (Kanto Chemical Co., Inc. (Japan)) IL containing 1.0-mol L^−1^ Li[TFSA] (Morita Chemical Industries Co., Ltd. (Japan)). The cell was assembled in an argon-filled glove box (VAC, OMNI-LAB, H_2_O, O_2_ < 1 ppm) and immediately placed on a sample holder in the *in situ* SEM system.

### *In situ* SEM experiments

We converted a conventional SEM system (S-3400N, Hitachi (Japan); VE-8800, Keyence (Japan)) into an *in situ* system by attaching feed-through terminals to examine the morphological and electrochemical behaviours of the Si anode. Both SE and BSE modes were used for the investigation. For the charge/discharge experiments, the CC/CV and CC modes were employed. The electrochemical experimental conditions were controlled with a potentiostat/galvanostat (VersaSTAT 4, Princeton Applied Research (USA)). All the electrochemical experiments were conducted in the chamber of the *in situ* SEM system, as described in our previous work^30^. *Ex situ* SEM of the Si anode after the charge/discharge experiments was conducted after rinsing with battery-grade DEC (Wako (Japan)) in an argon-filled glove box.

## Additional Information

**How to cite this article**: Chen, C.-Y. *et al. In situ* Scanning Electron Microscopy of Silicon Anode Reactions in Lithium-Ion Batteries during Charge/Discharge Processes. *Sci. Rep.* **6**, 36153; doi: 10.1038/srep36153 (2016).

**Publisher’s note:** Springer Nature remains neutral with regard to jurisdictional claims in published maps and institutional affiliations.

## Supplementary Material

Supplementary Movie 1

Supplementary Movie 2

Supplementary Movie 3

Supplementary Movie 4

Supplementary Movie 5

Supplementary Movie 6

Supplementary Information
