# Supplementary material for: In situ Scanning Electron Microscopy of Silicon Anode Reactions in Lithium-Ion Batteries during Charge/Discharge Processes
Source: Sci Rep. 2016 Oct 26;6:36153. doi: 10.1038/srep36153 (PMC5080607; doi:10.1038/srep36153)
Supplement: Supplementary Information [file srep36153-s7.docx]

***In situ* Scanning Electron Microscopy of Silicon Anode Reactions in Lithium-Ion Batteries during Charge/Discharge Processes**

Chih-Yao Chen,^1^ Teruki Sano,^1^ Tetsuya Tsuda,^1^ Koichi Ui,^2^ Yoshifumi Oshima,^3^ Masaki Yamagata,^4^ Masashi Ishikawa,^4^ Masakazu Haruta,^5^ Takayuki Doi,^5^ Minoru Inaba^5^ & Susumu Kuwabata^1^

^1^Department of Applied Chemistry, Graduate School of Engineering, Osaka University, 2-1 Yamada-oka, Suita, Osaka 565-0871, Japan

^2^Department of Frontier Materials and Function Engineering, Graduate School of Engineering, Iwate University, 4-3-5 Ueda, Morioka, Iwate 020-8551, Japan

^3^School of Materials Science, Japan Advanced Institute of Science and Technology, 1-1 Asahidai, Nomi, Ishikawa 923-1292, Japan

^4^Department of Chemistry and Materials Engineering, Kansai University, 3-3-35 Yamate-cho, Suita, Osaka 564-8680, Japan

^5^Department of Molecular Chemistry and Biochemistry, Doshisha University, 1-3 Tatara Miyakodani, Kyotanabe, Kyoto 610-0321, Japan

Correspondence and requests for materials should be addressed to T.T. (e-mail: ttsuda@chem.eng.osaka-u.ac.jp) or S.K. (e-mail: kuwabata@chem.eng.osaka-u.ac.jp)

**Supplementary Information**

Movie legends p. 2

Supplementary Figures 1–4 p. 4

**Movie legends**

**Movie 1 | *In situ* SEM images of a Si microparticle anode during the 2nd charge process.** The charge process was conducted in constant current and constant voltage (CC/CV) mode with cut-off voltages ranging between –3.88 V and –2.40 V (*vs*. LiCoO_2_). The CC rates for charge and discharge were 0.12 C. The video clip plays at 640 × speed.

**Movie 2 | *In situ* SEM images of a Si microparticle anode during the 2nd discharge process.** The discharge process was conducted in constant current and constant voltage (CC/CV) mode with cut-off voltages ranging between –3.88 V and –2.40 V (*vs*. LiCoO_2_). The CC rates for charge and discharge were 0.12 C. The video clip plays at 640 × speed.

**Movie 3 | *In situ* SEM images of a Si thin flake anode during the 3rd charge process.** The charge process was conducted in constant current and constant voltage (CC/CV) mode with cut-off voltages ranging between –3.88 V and –2.40 V (*vs*. LiCoO_2_). The CC rates for charge and discharge were 0.50 C. The video clip plays at 640 × speed.

**Movie 4 | *In situ* SEM images of a Si thin flake anode during the 3rd discharge process.** The discharge process was conducted in constant current and constant voltage (CC/CV) mode with cut-off voltages ranging between –3.88 V and –2.40 V (*vs*. LiCoO_2_). The CC rates for charge and discharge were 0.50 C. The video clip plays at 640 × speed.

**Movie 5 | *In situ* SEM and BSE images of a Si thin flake anode during the 2nd discharge process.** The charge process was conducted in constant current and constant voltage (CC/CV) mode with cut-off voltages ranging between –3.88 V and –2.40 V (*vs*. LiCoO_2_). The CC rates for charge and discharge were 0.50 C. The video clip plays at 640 × speed.

**Movie 6 | *In situ* SEM and BSE images of a Si thin flake anode during the 3rd charge process.** The discharge process was conducted in constant current and constant voltage (CC/CV) mode with cut-off voltages ranging between –3.88 V and –2.40 V (*vs*. LiCoO_2_). The CC rates for charge and discharge were 0.50 C. The video clip plays at 640 × speed.


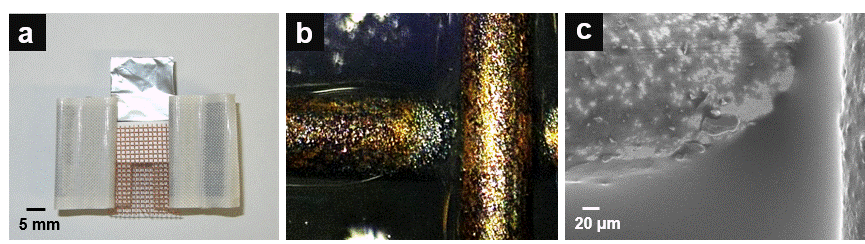


**Supplementary Figure 1 | A typical LIB cell used for *in situ* SEM. a**, A photograph of the LIB cell. **b**, A digital microscope image of a Si microparticle electrode. **c**, A SEM image recorded in the same area shown in Supplementary Fig. 1b.


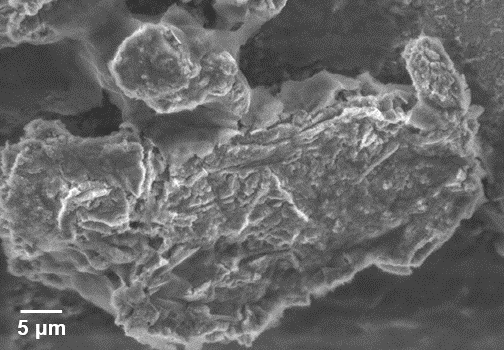


**Supplementary Figure 2 | An *ex situ* SEM image of the Si particle shown in Fig. 2g after rinsing with DEC.**


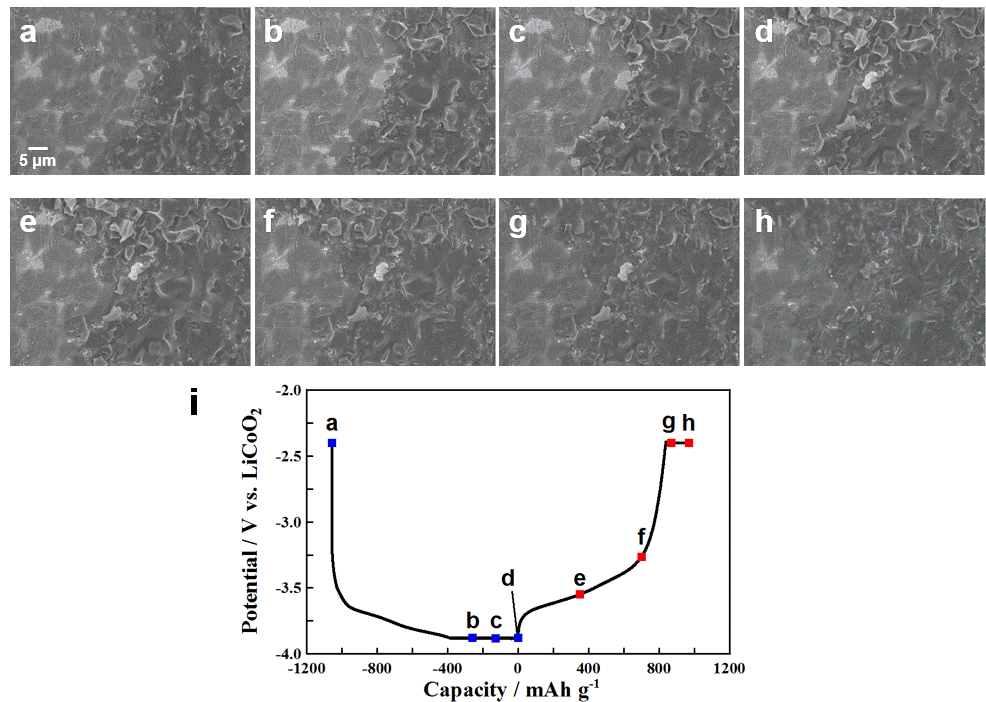


**Supplementary Figure 3 | *In situ* SEM images of the morphology variation of Si thin flakes during charging and discharging in the 3rd cycle.** The charge/discharge curve of a Si thin flake anode measured in constant current and constant voltage (CC/CV) mode with cut-off voltages ranging between –3.88 V and –2.40 V (*vs*. LiCoO_2_). The CC rates for charge and discharge were 0.50 C. These images were captured at the points indicated by **a-h** in Supplementary Fig. 3i. **i**, The charge/discharge curve during the *in situ* SEM observation.


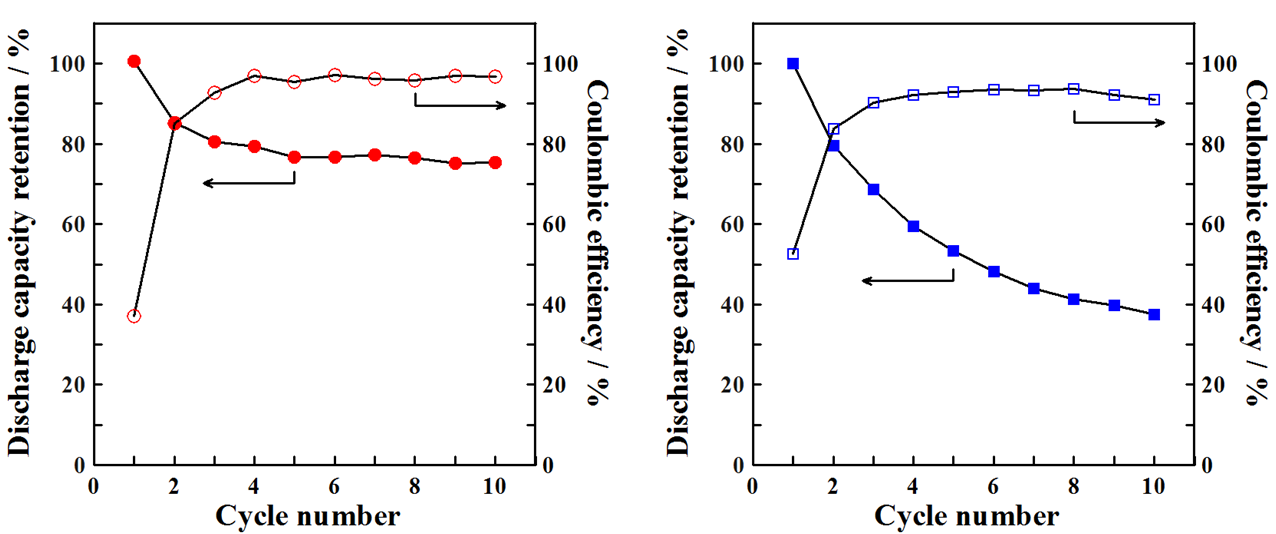


**Supplementary Figure 4 | Discharge capacity retention and coulombic efficiency of LIB cells used for *in situ* SEM.** Si thin flake and Si nanoparticle aggregate electrodes are shown as red circles and blue squares, respectively.
